# Integrating augmented intelligence and artificial intelligence into primary healthcare

**DOI:** 10.51866/lte.25.01

**Published:** 2025-10-07

**Authors:** S Kaviyarasan Sailin @ Stalin

**Affiliations:** 1 President-Elect, Academy of Family Physicians of Malaysia, Unit 3.11, Level 3, Medical Academies Malaysia, 5, Jalan P8H, Presint 8, Putrajaya, Wilayah Persekutuan Putrajaya, Malaysia. Email: drkavi11@gmail.com

**Keywords:** Artificial intelligence, Augmented intelligence, Primary health care, Education

**Dear editor,**

*‘The prevailing vision for the future of healthcare is not one of AI replacing clinicians, but of AI and humans working together — a principle central to the concept of augmented intelligence’.*

-IBM Watson Health

The 21st century is witnessing a rapid evolution in the field of technology. Just as Edward Jenner’s landmark discovery of the smallpox vaccine in the late 18th century launched a medical revolution in the field of science, artificial intelligence (AI) is now impacting every sector, bringing immense changes in technology, education and medicine. In primary healthcare, AI scribes are reshaping clinical consultations by automating documentation, improving accuracy, enhancing efficiency and reducing administrative burden. AI is now blurring the frontiers between human and machine interactions, raising significant ethical issues in patients’ data protection, consent, bias, discrimination and social impact, particularly in job retrenchment. This fundamental shift compels healthcare providers, educators and policymakers to rethink the design, delivery and governance of primary healthcare.

## Conception of AI

In 1950, Alan Turing, an English mathematician and computer pioneer, posed the question, ‘Can machines think?’ In his paper, ‘Computing machinery and intelligence’, he laid the seed for AI. In 1956, 2 years after the death of Alan Turing, John McCarthy, a professor at Dartmouth College, organised a summer workshop to explore the concept of thinking machines, coining the term ‘artificial intelligence’.^1^ Since then, the concept of AI has become an integral part of numerous sectors, from simple applications in small- and medium-sized enterprises to advanced systems in cloud computing, education and primary healthcare.

## AI

The world is currently in the midst of the Fourth Industrial Revolution, a period marked by rapid transformation as digital technologies such as cloud computing, the Internet of Things and AI are embraced. The convergence of these innovations - alongside groundbreaking medical advances, such as the Neuralink brain implant - is poised to deliver benefits the world has never seen before.

Across various sectors, automation and robotics are enhancing efficiency and productivity. In this new era of AI, collaborative robots^2^ - commonly known as ‘cobots’ - are being developed to work harmoniously with humans. These technologies foster synergistic work environments that improve productivity, safety and operational efficiency by leveraging data and connectivity.

AI is making technology and services more available, accessible and transferable to clinicians, researchers, educators, institutions and stakeholders across the healthcare ecosystem and supply chain. This advancement is unlocking countless benefits and reshaping the future of healthcare delivery.

## What is augmented intelligence (AuI)?

In 1962, Douglas Engelbart discussed a framework for augmenting human intelligence. He described augmentation as ‘increasing the capability of a man to approach a complex problem situation, to gain comprehension to suit his particular needs and to derive solutions to problems’^3^

AuI is the use of technology to enhance a human’s ability to execute tasks, perform analysis and make decisions.^4^ In AuI, machine learning and AI systems are commonly used in an assistive role to help humans in a task, as opposed to replacing them.^4^ In practice, most AI applications today support AuI since humans are also involved in the processes of developing and implementing AI, providing context, improving accuracy and ensuring its safety. AuI involves the use of technology to enhance human intelligence, while AI is defined as the simulation of human intelligence by machines. In practice, they are highly complementary and used to augment human intelligence rather than replace it.^4^

## AI in primary healthcare

*AI is not going to replace doctors — but doctors who use AI will replace those who don’t’.*

- AMA President Jesse M. Ehrenfeld, MD, MPH

AI has great potential in general practice, but as these technologies become more advanced, the risks that they pose must be carefully mitigated.^4^ Primary healthcare is beginning to integrate AI in its system to improve accuracy in documentation, enhance efficiency in practice and reduce administrative burden and physician burnout. The COVID-19 pandemic accelerated the adoption of AI, requiring physical distancing and isolation, which shifted patient care through digitisation and telehealth.

Readiness is essential to tap into the innumerable benefits of AI in primary healthcare, some of which include the following:

1. **Al-powered scribing:** Tools that record the clinical consultation between the physician and patient, transcribe the conversation, generate clinical notes and integrate them with electronic medical records.^5^
2. **Streamlined administrative tasks:** AI supports seamless documentation, medical record keeping, data analytics and billing, enhancing efficiency.^5^
3. **Improved patient-doctor interaction:** With reduced documentation time, clinicians can focus more on communication, fostering stronger therapeutic relationships and higher patient satisfaction.
4. **Reduction in physician burnout:** By automating routine tasks, AI scribe tools alleviate mental load and enhance job satisfaction.
5. **Standardisation of care:** Care is standardised across various healthcare settings, reducing urbanrural gaps.^5^
6. **Enhanced diagnostics:** In secondary and tertiary care, AI is being used to analyse medical imaging (e.g. radiographs, mammograms, MRIs and CT scans), enabling earlier and more accurate diagnoses.

## AI in education and training

AI technologies are increasingly being applied to enhance education and training across a wide range of subjects, including languages, science, technology, engineering, mathematics and medicine, transforming how pedagogy and learning are delivered worldwide.^6^ This evolving landscape of education, the integration of AI, represents a transformative shift, stipulating a new era in learning and teaching methodologies.^6^ Its role extends beyond traditional teaching methods, offering personalised learning experiences and supporting a diverse range of educational needs. It enhances educational processes, developing essential skills such as computational and critical thinking, intricately linked to machine learning and educational robotics.^6^ It also requires a paradigm shift in how education is approached in the AI era, moving beyond traditional methods to embrace more dynamic, interactive and student-centred learning environments.^7^ Learners, teachers, mentors and educational institutions have to quickly embrace AI in education more now than ever before.

Primary healthcare providers need to keep pace with the rapid development in the field of AI, learning new skills and applications. They must explore the prospective influence of AI on medical education, postgraduate training and continuing education of attending physicians, mentors and consultants.^8^ The emergence of chatbots, particularly those powered by advanced large language models such as GPT-4 and Google BARD, has sparked renewed discussions about the integration of AI into medical education, hospital operations and the broader healthcare ecosystem.^9,10^ Strengthening collaborative efforts between policymakers, healthcare providers and AI developers will be essential in shaping an equitable, efficient and patient-centred AI-driven primary healthcare system.^11^

The World Medical Association advocates the need to update medical curricula to ensure healthcare professionals are well-versed in AI’s benefits and risks in healthcare.^12^ In line with this, a significant majority of surveyed medical students and doctors from countries including the USA, the UK, Germany and Turkiye have expressed their approval of incorporating structured AI-related training courses into medical curricula.^12^

## Challenges with AI in education and training

Although AI demonstrates significant potential, several challenges must be addressed before its widespread implementation in practice, including the following:

1. **Generation of incorrect or misleading outputs,** which can undermine the reliability of AI tools.
2. **Provision of inaccurate or incomplete medical treatment information**, which may lead to serious consequences.^12^
3. **Lack of an established accountability framework** to address the implications of AI-generated content.^12^
4. **Limitations in data generation**, particularly when reliant on user-provided inputs, and difficulty in responding to complex or unconventional queries.^13^
5. **Risk of over-reliance on AI**, which may inhibit critical thinking and clinical reasoning, potentially overlooking inherent risks associated with AI tools.^14^

## Conclusion

AI is reshaping primary healthcare by improving clinical consultations, streamlining documentation, enhancing diagnostic accuracy and reducing administrative burden, while AuI promotes a synergistic relationship between clinicians and AI. Both present challenges related to data privacy, ethical considerations and workplace adaptation and are poised to revolutionise various aspects of medical education and training through a paradigm shift from traditional pedagogy to AI-integrated educational models. With strategic implementation and a focus on responsible innovation, AI can significantly elevate the quality, efficiency and accessibility of primary healthcare. AI should be embraced with optimism and responsibility to advance the future of primary healthcare.

*‘The future of AI is in our hands’.*

- Tim Cook, CEO of Apple
